# P Wave Dispersion and Maximum P Wave Duration Are Independently Associated with Rapid Renal Function Decline

**DOI:** 10.1371/journal.pone.0042815

**Published:** 2012-08-27

**Authors:** Ho-Ming Su, Wei-Chung Tsai, Tsung-Hsien Lin, Po-Chao Hsu, Wen-Hsien Lee, Ming-Yen Lin, Szu-Chia Chen, Chee-Siong Lee, Wen-Chol Voon, Wen-Ter Lai, Sheng-Hsiung Sheu

**Affiliations:** 1 Division of Cardiology, Department of Internal Medicine, Kaohsiung Medical University Hospital, Kaohsiung Medical University, Kaohsiung, Taiwan; 2 Division of Nephrology, Department of Internal Medicine, Kaohsiung Medical University Hospital, Kaohsiung Medical University, Kaohsiung, Taiwan; 3 Department of Internal Medicine, Kaohsiung Municipal Hsiao-Kang Hospital, Kaohsiung Medical University, Kaohsiung, Taiwan; 4 Faculty of Medicine, College of Medicine, Kaohsiung Medical University, Kaohsiung, Taiwan; University of Sao Paulo Medical School, Brazil

## Abstract

The P wave parameters measured by 12-lead electrocardiogram (ECG) are commonly used as noninvasive tools to assess for left atrial enlargement. There are limited studies to evaluate whether P wave parameters are independently associated with decline in renal function. Accordingly, the aim of this study is to assess whether P wave parameters are independently associated with progression to renal end point of ≥25% decline in estimated glomerular filtration rate (eGFR). This longitudinal study included 166 patients. The renal end point was defined as ≥25% decline in eGFR. We measured two ECG P wave parameters corrected by heart rate, i.e. corrected P wave dispersion (PWdisperC) and corrected P wave maximum duration (PWdurMaxC). Heart function and structure were measured from echocardiography. Clinical data, P wave parameters, and echocardiographic measurements were compared and analyzed. Forty-three patients (25.9%) reached renal end point. Kaplan-Meier curves for renal end point-free survival showed PWdisperC > median (63.0 ms) (log-rank *P* = 0.004) and PWdurMaxC > median (117.9 ms) (log-rank *P*<0.001) were associated with progression to renal end point. Multivariate forward Cox-regression analysis identified increased PWdisperC (hazard ratio [HR], 1.024; *P* = 0.001) and PWdurMaxC (HR, 1.029; *P* = 0.001) were independently associated with progression to renal end point. Our results demonstrate that increased PWdisperC and PWdurMaxC were independently associated with progression to renal end point. Screening patients by means of PWdisperC and PWdurMaxC on 12 lead ECG may help identify a high risk group of rapid renal function decline.

## Introduction

The risk for progression of renal function is contributed to traditional risk factors such as hypertension, diabetes, and dyslipidemia and non-traditional risk factors including cardiovascular disease [1], [2]. Progressive decline in renal function was significantly associated with high cardiovascular morbidity and mortality, independent of baseline renal function [3], [4]. Therefore, identifying the patients with rapid renal function progression for aggressive treatment interventions is important in disease attenuation and prolonged survival.

Recently, echocardiographic measures of left ventricular function and structure as well as left atrial size have been reported to predict adverse renal outcomes [5]–[7]. We have reported that increased left atrial diameter and decreased left ventricular ejection fraction (LVEF) were independently associated with faster renal function decline [5], [6]. Besides, Furukawa et al. [7] found that left atrial volume index (LAVI) was an independent risk factor for the period of time before dialysis in patients with chronic kidney disease (CKD) stages 4–5. This implied that patients with an increased left atrial diameter might have a rapid renal function decline and adverse renal outcome. However, left atrial size is frequently evaluated using echocardiography, which may preclude its application if echocardiography or experienced operators are not available. The parameters obtained from 12-lead electrocardiogram (ECG) such as frontal T wave axis deviation and QT interval prolongation are reported to be significantly associated with renal insufficiency [8], [9]. The 12-lead ECG is also a simple and cheap noninvasive clinical tool to access left atrial enlargement [10]. There were several ECG parameters for the detection of left atrial enlargement, such as P wave morphology, P wave dispersion, P wave duration, terminal negative deflection in precordial lead V1, and P wave area [11]–[18]. Furthermore, P wave parameters were independently associated with increased risks for atrial fibrillation and recurrent transient ischemic attacks [19], [20]. However, there are limited studies to evaluate whether P wave parameters are independently associated with decline in renal function. Accordingly, the aim of this study is to assess whether P wave parameters are independently associated with progression to renal end point of ≥25% decline in estimated glomerular filtration rate (eGFR).

## Methods

### Study Patients

Study subjects were included from a group of patients who arranged for echocardiographic examinations at Kaohsiung Municipal Hsiao-Kang Hospital. Patients with significant aortic or mitral valve diseases, atrial fibrillation, hemodialysis, and inadequate image visualization were excluded. We did not include all patients consecutively because 12-lead surface ECG must be measured within 5 minutes after the completion of an echocardiographic examination. In addition, those patients with follow-up time less than 6 months and less than three eGFR measurements during the follow-up period were excluded to avoid incomplete observation of change in renal function. Study patients were enrolled from January 2007 to September 2007 and they were followed until May 2010. Finally, a total of one hundred and sixty-six patients (mean age 57.4±13.7 years, 94 males/72 females) were included.

### Ethics Statement

The study protocol was approved by the institutional review board of the Kaohsiung Medical University Hospital (KMHK-IRB-A96010863). Informed consents have been obtained in written form from patients and all clinical investigation was conducted according to the principles expressed in the Declaration of Helsinki. The patients gave consent for the publication of the clinical details.

### Assessment of P Wave Parameters

Within 5 minutes after the completion of echocardiographic examination, the standard 12-lead surface ECG (25-mm/s, 1-mV/cm, and 100-Hz) was recorded. Quantitative assessments were performed by using image analysis software system (Image Tool 3.0). We used two P wave parameters, i.e. corrected P wave maximum duration (PWdurMaxC) and corrected P wave dispersion (PWdisperC), for assessing their correlation with decline in renal function. The P wave dispersion was calculated by the difference between maximum P wave duration and minimum P wave duration [21]. Both the P wave measurements were corrected for heart rate by Bazet
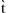
s formula, i.e. the corrected P wave parameters was equal to P wave parameters/(RR)^1/2^ [22].

### Evaluation of Cardiac Structure and Function

The echocardiographic examination was performed by one experienced sonographer using transthoracic echocardiography (Vivid 7; General Electric Medical Systems, Horten, Norway), with the participant respiring quietly in the left decubitus position. Two-dimensional and two-dimensionally guided M-mode images were recorded from the standardized views. Left ventricular mass was calculated using Devereux-modified method [23]. Left ventricular mass index (LVMI) was calculated by dividing left ventricular mass by body surface area. Left ventricular hypertrophy (LVH) was defined as suggested by the 2007 European Society of Hypertension/European Society of Cardiology guidelines [24]. The Doppler sample volume was placed at the tips of the mitral leaflets to get the left ventricular inflow waveforms from the apical 4-chamber view. All sample volumes were positioned with ultrasonic beam alignment to flow. Pulsed tissue Doppler imaging was obtained with the sample volume placed at the lateral corner of the mitral annulus from the apical 4-chamber view. The wall filter settings were adjusted to exclude high-frequency signals and the gain was minimized. LVEF was measured by the modified Simpson’s method. The left atrial volume was measured by the biplane area–length method [25]. Apical 4- and 2-chamber views were obtained to determine the left atrial area and length (from the middle of the plane of the mitral annulus to the posterior wall). The maximal left atrial chamber area and length were measured before mitral valve opening, excluding the left atrial appendage and pulmonary veins. LAVI was calculated by dividing left atrial volume by body surface area. The raw ultrasonic data were recorded and analyzed offline by a cardiologist, blinded to the other data, using EchoPAC software (GE Medical Systems). The echocardiographic data were obtained from three consecutive beats and then the data were averaged to give the mean value for later analysis.

### Collection of Demographic, Medical, and Laboratory Data

Demographic and medical data including age, gender, smoking history (ever *versus* never), and comorbid conditions were obtained from medical records or interviews with patients. The body mass index (BMI) was calculated as the ratio of weight in kilograms divided by square of height in meters. Laboratory data were measured from fasting blood samples using an autoanalyzer (Roche Diagnostics GmbH, D-68298 Mannheim COBAS Integra 400). Serum creatinine was measured by the compensated Jaffé (kinetic alkaline picrate) method in a Roche/Integra 400 Analyzer (Roche Diagnostics, Mannheim, Germany) using a calibrator traceable to isotope-dilution mass spectrometry [26]. The value of eGFR was calculated using the 4-variable equation in the Modification of Diet in Renal Disease (MDRD) study [27]. Proteinuria was examined by dipsticks (Hema-Combistix, Bayer Diagnostics). A test result of 1+ or more was defined as positive. Blood and urine samples were obtained within 1 month of enrollment. In addition, information regarding patient antihypertensive medications use during the study period was obtained from medical records.

### Definition of Renal End Point

The renal end point was defined as ≥25% decline in eGFR since enrollment [28]. In patients reaching renal end point, renal function data were censored. The patients who did not reach renal end points were followed until May 2010.

### Statistical Analysis

Statistical analysis was performed using SPSS version 15.0 (SPSS Inc., Chicago, IL, USA) for windows. Data are expressed as percentages, mean ± standard deviation or median (25^th^–75^th^ percentile) for triglyceride and number of serum creatinine measurements. The differences between groups were checked by Chi-square test for categorical variables and by independent t-test for continuous variables. Time to renal end point of ≥25% decline in eGFR and covariates of risk factors were modeled using the Cox proportional hazards model. The relationship between two normally distributed continuous variables was assessed by Pearson’s correlation. Spearman’s correlation was used to assess the correlation between continuous variables without normal distribution, including BMI, fasting glucose, triglyceride and baseline eGFR. Significant variables in univariate analysis were selected for multivariate forward analysis. A difference was considered significant if the *P* value was less than 0.05.

## Results

A total of one hundred and sixty-six patients were included. The mean age was 57.4±13.7 years and there were 94 males and 72 females. The average number of serum creatinine measurements during the follow-up period was 5 (25^th^–75^th^ percentile: 4–8.5) times. The values of PWdisperC and PWdurMaxC were 67.0±24.9 and 119.5±24.8 ms, respectively. The comparison of baseline characteristics between patients with and without renal end point of ≥25% decline in eGFR is shown in Table 1. Compared with patients without renal end point, patients with renal end point were found to have higher prevalence of smoking history, higher prevalence of diabetes mellitus (DM), lower albumin, higher fasting glucose, lower hematocrit, lower baseline eGFR, higher uric acid, higher prevalence of proteinuria, higher PWdisperC, and higher PWdurMaxC.

**Table 1:** Comparison of baseline characteristics between patients with and without renal end point of ≥25% decline in eGFR.

Characteristics	All patients (n = 166)	Patients without renal end point (n = 123)	Patients with renal end point (n = 43)
Age (year)	57.4±13.7	56.4±12.8	60.3±15.8
Male gender (%)	56.6	53.7	65.1
Smoking history (%)	35.7	28.1	57.5*
Diabetes mellitus (%)	28.3	17.1	60.5**
Hypertension (%)	64.5	63.4	67.4
Coronary artery disease (%)	20.5	19.5	23.3
Systolic BP (mmHg)	136.9±21.1	137.6±21.0	134.9±21.3
Diastolic BP (mmHg)	80.2±11.8	80.3±11.4	79.8±13.0
Pulse pressure (mmHg)	56.7±15.3	57.3±16.0	55.0±13.0
Body mass index (kg/m^2^)	25.6±4.0	26.0±4.1	24.6±3.7
Laboratory parameters			
Albumin (g/dL)	4.03±0.45	4.13±0.36	3.77±0.54**
Fasting glucose (mg/dL)	119.3±47.7	113.8±43.6	135.2±55.3*
Triglyceride (mg/dL)	140 (92.5–198)	132.5 (90.25–187)	156 (98.5–277)
Total cholesterol (mg/dL)	205.2±82.6	205.5±82.7	204.3±83.2
Hematocrit (%)	41.9±5.3	43.1±4.3	38.5±6.4**
Baseline eGFR (mL/min/1.73 m^2^)	57.0±17.2	61.1±13.3	45.4±21.4**
Uric acid (mg/dL)	6.9±2.2	6.4±1.8	8.4±2.4**
Proteinuria (%)	23.7	14.8	48.8**
Antihypertensive medication use (%)	79.5	77.2	86.0
Electrocardiogram data			
PWdisperC (ms)	67.0±24.9	64.0±24.4	75.4±24.7*
PWdurMaxC (ms)	119.5±24.8	117.0±24.5	126.4±24.7*

Abbreviations. BP, blood pressure; eGFR, estimated glomerular filtration rate; ACEI, angiotensin converting enzyme inhibitor; ARB, angiotensin II receptor blocker; PWdisperC, corrected P wave dispersion; PWdurMaxC, corrected P wave maximum duration.

* *P*<0.05,

** *P*<0.001 compared with patients without renal end point.

### Risk of Progression to Renal End Point of ≥25% Decline in eGFR

The mean follow-up period was 30.9±12.2 months. During the period of follow-up, forty-three patients (25.9%) reached renal end point. Three patients entering hemodialysis and 6 patients with mortality were recorded. However, all of them reached the renal end point earlier than they received dialysis or died. Table 2 shows a Cox proportional hazards regression analysis for progression to renal end point of ≥25% decline in eGFR. The Cox proportional hazards univariate regression analysis showed that a smoking history, DM, low albumin, high fasting glucose, high triglyceride, low hematocrit, low baseline eGFR, high uric acid, proteinuria, and increased PWdisperC and PWdurMaxC were associated with an increase in progression to renal end point. We performed two multivariate forward analyses. In the first multivariate analysis (model 1: covariates included significant variables in the univariate analysis except PWdurMaxC), low albumin, low hematocrit, high uric acid, and increased PWdisperC (hazard ratio [HR], 1.024; 95% confidence interval [CI], 1.009 to 1.039; *P* = 0.001) were independently associated with progression to renal end point. In the second multivariate analysis (model 2: covariates included significant variables in the univariate analysis except PWdisperC), low albumin, low hematocrit, high uric acid, and increased PWdurMaxC (HR, 1.029; 95% CI, 1.011 to 1.047; *P* = 0.001) were independently associated with progression to renal end point. Two equations were created to predict the probability of significant reduction in renal function. Equation 1: log(HR)  =  −1.932 × (albumin) − 0.202 × (hematocrit) + 0.180 × (uric acid) + 0.023 × PWdisperC; Equation2: log(HR)  =  −1.958 × (albumin) − 0.207 × (hematocrit) + 0.184 × (uric acid) + 0.029 × PWdurMaxC. In addition, the interaction between PWdisperC and uric acid to renal end point was statistically significant (OR, 1.002; 95% CI, 1.001 to 1.003; *P*<0.001). Similarly, the interaction between PWdurMaxC and uric acid to renal end point was also statistically significant (OR, 1.001; 95% CI, 1.001 to 1.002; *P*<0.001).

**Table 2:** Predictors of progression to renal end point (≥25% decline in eGFR) using Cox proportional hazards model.

Parameter	Univariate		Multivariate (forward)				
	HR (95% CI)	*P*	HR (95% CI)		*P*	HR (95% CI)	*P*
			Model 1			Model 2	
Age (per 1 year)	1.020 (0.996–1.044)	0.099	–	–		–	–
Male *versus* female	1.543 (0.824–2.891)	0.175	–	–		–	–
Smoking (ever *versus* never)	2.830 (1.511–5.300)	0.001	–	–		–	–
Diabetes mellitus	5.277 (2.857–9.749)	<0.001	–	–		–	–
Hypertension	1.128 (0.596–2.135)	0.711	–	–		–	–
Coronary artery disease	1.235 (0.608–2.509)	0.559	–	–		–	–
Systolic BP (per 1 mmHg)	0.995 (0.980–1.010)	0.482	–	–		–	–
Diastolic BP (per 1 mmHg)	0.998 (0.972–1.025)	0.905	–	–		–	–
Pulse pressure (per 1 mmHg)	0.991 (0.971–1.011)	0.385	–	–		–	–
Body mass index (per 1 kg/m^2^)	0.924 (0.852–1.003)	0.059	–	–		–	–
Laboratory parameters							
Albumin (per 1 g/dL)	0.366 (0.237–0.566)	<0.001	0.145 (0.063–0.334)	<0.001		0.141 (0.061–0.328)	<0.001
Fasting glucose (per 1 mg/dL)	1.007 (1.002–1.012)	0.005	–	–		–	–
Triglyceride (per log 1 mg/dL)	2.969 (1.216–7.253)	0.017	–	–		–	–
Total cholesterol (per 1mg/dL)	1.000 (0.996–1.004)	0.912	–	–		–	–
Hematocrit (per 1%)	0.867 (0.824–0.912)	<0.001	0.817 (0.758–0.881)	<0.001		0.813 (0.752–0.878)	<0.001
Basline eGFR (per 1 mL/min/1.73 m^2^)	0.948 (0.932–0.965)	<0.001	–	–		–	–
Uric acid (per 1 mg/dL)	1.373 (1.220–1.545)	<0.001	1.197 (1.036–1.383)	0.015		1.202 (1.037–1.394)	0.015
Proteinuria	4.092 (2.208–7.585)	<0.001	–	–		–	–
Antihypertensive medication use	1.671 (0.705–3.960)	0.244	–	–		–	–
Electrocardiogram data							
PWdisperC (per 1 ms)	1.017 (1.005–1.028)	0.005	1.024 (1.009–1.039)	0.001			
PWdurMaxC (per 1 ms)	1.016 (1.004–1.029)	0.009				1.029 (1.011–1.047)	0.001

Values express as hazard ratios (HR) and 95% confidence interval (CI). The other abbreviations are the same as in Table 1.

**Figure 1:**
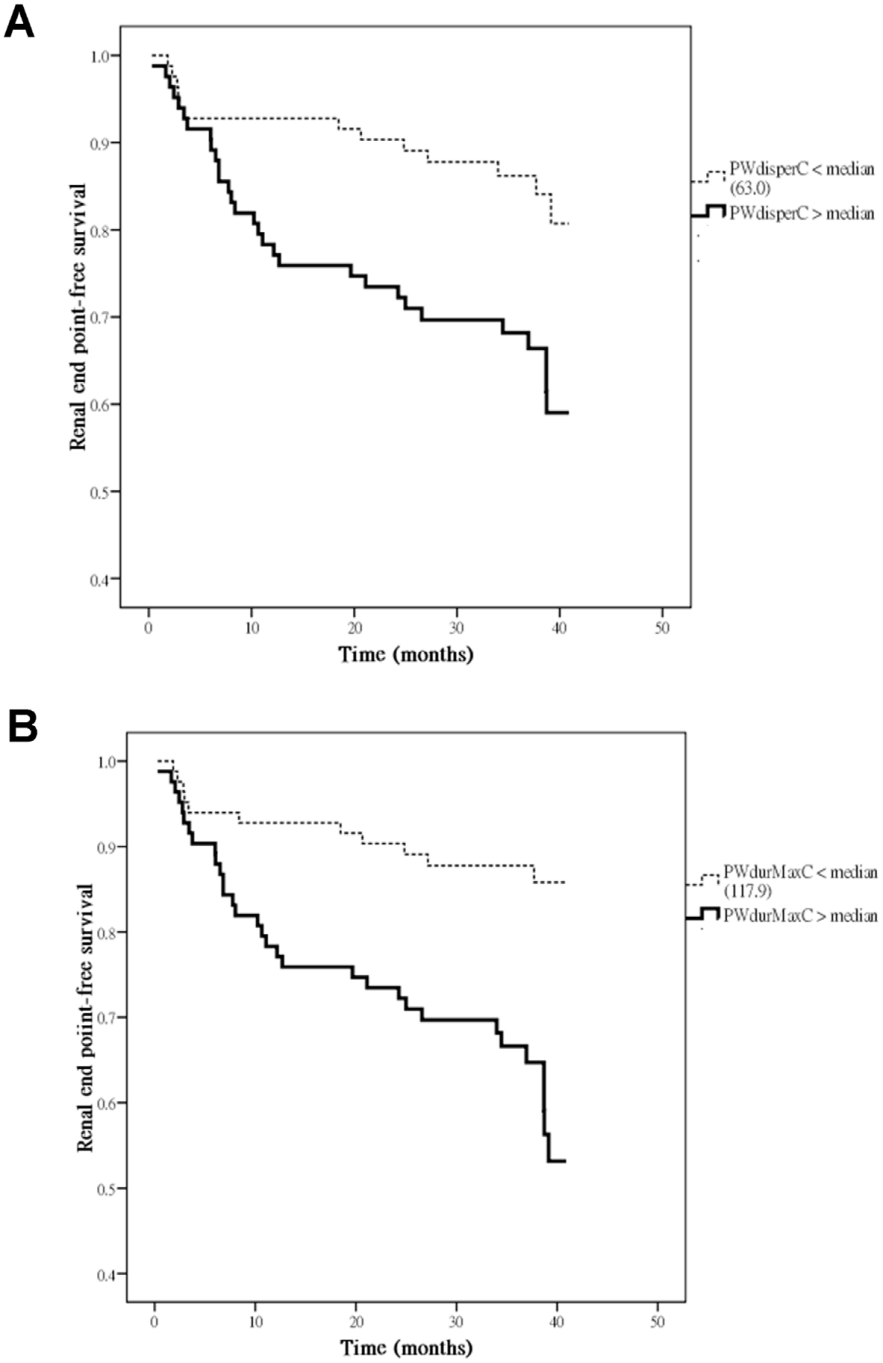
Kaplan-Meier analysis of renal end point-free survival according to PWdisperC ≥ median (63.0 ms) or < median (log-rank *P* = 0.004) (A) and PWdurMaxC ≥ median (117.9 ms) or < median (log-rank *P*<0.001) (B).

Figure 1 illustrated the Kaplan-Meier curves for renal end point-free survival in all patients subdivided according to PWdisperC ≥ median (63.0 ms) or < median (log-rank *P* = 0.004) (A) and PWdurMaxC ≥ median (117.9 ms) or < median (log-rank *P*<0.001) (B).

### Determinants of PWdisperC and PWdurMaxC

In the univariate analysis, PWdisperC had a significantly positive correlation with male, a smoking history, fasting glucose, and uric acid and negative correlation with systolic blood pressure, pulse pressure and albumin. After the multiple forward analysis, PWdisperC was correlated independently with uric acid (β = 0.281, *P* = 0.002). In addition, PWdurMaxC had a significantly positive correlation with male, a smoking history, fasting glucose, hematocrit, uric acid, and proteinuria and negative correlation with age, pulse pressure, and albumin in the univariate analysis. After the multiple forward analysis, PWdurMaxC was correlated independently with uric acid (β = 0.308, *P* = 0.001).

### Correlation between P Wave Parameters and Echocardiographic Data

PWdisperC was correlated with LVMI (*r* = 0.264, *P* = 0.001), LVH (*P* = 0.018), LVEF (*r* = −0.286, *P*<0.001), transmitral E wave velocity (E)/early diastolic mitral velocity (Ea) ratio (*r* = 0.198, *P* = 0.011), and LAVI (*r* = 0.275, *P*<0.001). In addition, PWdurMaxC was correlated with LVMI (*r* = 0.297, *P*<0.001), LVH (*P* = 0.002), LVEF (*r* = −0.346, *P*<0.001), E/Ea ratio (*r* = 0.201, *P* = 0.010), and LAVI (*r* = 0.378, *P*<0.001).

## Discussion

In the present study, we evaluated the association of P wave parameters with the progression to renal end point of ≥25% decline in eGFR. We found that PWdisperC >63.0 ms and PWdurMaxC >117.9 ms were associated with progression to renal end point. In addition, increased PWdisperC and PWdurMaxC were associated with worse echocardiographic profiles and independently associated with reaching the renal end point.

Cardiovascular dysfunction is associated with declining renal function [4], [29], [30]. A general definition of cardiorenal syndrome is disorders of the heart and kidneys whereby acute or chronic dysfunction in one organ may induce acute or chronic dysfunction of the other [31]–[33]. The mechanisms of progressive renal function decline in patients with cardiac abnormalities are multi-factorial including chronic renal hypoperfusion, subclinical inflammation, endothelial dysfunction, accelerated atherosclerosis, increased renal vascular resistance, systemic neurohormonal factors, pharmacotherapies and anemia [31], [34]. Paoletti et al. [35] studied the role of LVH in prediction of progression to dialysis in 144 patients with CKD stage 3–4. They found increased LVMI was independently associated with progression to dialysis and combined end points of dialysis or death. They explained their findings by the same pathogenetic factors responsible for kidney damage and increase in both left ventricular wall thickness and internal dimension. Besides, Shlipak et al. [4] had demonstrated decreased left ventricular systolic function was an independent predictor of rapid renal function decline (defined by an annual eGFR loss >3 ml/min/1.73 m^2^) in the elderly. Furukawa et al. [7] also found LAVI was an independent risk factor for the period of time before dialysis. Recently, we had reported that concentric LVH, increased left atrial diameter, and decreased LVEF were associated with adverse renal outcomes [5], [6]. The P wave parameters measured by 12-lead ECG were reported to be useful tools for accessing risks of left atrial enlargement, LVH, left ventricular diastolic dysfunction, and atrial arrhythmia [14], [15], [17], [18], [36]–[39]. Our study also revealed that PWdisperC and PWdurMaxC were correlated with left atrial enlargement, LVH, and left ventricular systolic and diastolic dysfunction, which was consistent with previous findings [36], [39]. Left ventricular systolic and diastolic dysfunction and increased left atrial size have been reported to be correlated with adverse renal outcomes [5]–[7]. Hence, the association of PWdisperC and PWdurMaxC with LVEF, E/Ea, and LAVI in our study might partially explain the correlation of PWdisperC and PWdurMaxC with rapid renal function decline.

Cicoira M et al. [40] evaluated the impact of elevated uric acid levels on cardiac function in 150 patients with dilated cardiomyopathy. They found uric acid level correlated significantly with E, E/transmitral A wave velocity, E-wave deceleration time, and restrictive mitral filling pattern, which were markers of diastolic dysfunction [40]. Hyperuricema is highly prevalent in renal insufficiency, which may account for the decreased renal excretion of uric acid when renal function declines and the association of hyperuricemia with various risk factors for renal insufficiency, such as hypertension, DM, and left ventricular systolic dysfunction [41]–[43]. Furthermore, experimental hyperuricemia could result in renal injury [44]. Consistent with the experimental model, hyperuricemia in humans was proven to be associated with adverse renal outcomes [6], [45]. Our results demonstrated that increased PWdisperC and PWdurMaxC were independently associated with elevated uric acid level. The close association of P wave parameters and serum uric acid might be an expression of renal hypoperfusion caused by reduced left ventricular systolic function. In addition, the correlation between increased PWdisperC and PWdurMaxC and renal end point might partially explained by elevated serum uric acid. We also found the interaction between the P wave parameters and uric acid to renal end point was statistically significant. Hence, hyperuricemia and increased P wave parameters might have a synergic effect on the risk of rapid renal progression.

In the present study, eGFR was calculated using the 4-variable MDRD equation [27]. Some shortcomings of MDRD equation should be addressed. The MDRD equation is derived from patients with CKD and in potential kidney donors. Therefore, it may not be accurate in normal renal function, such as patient with type 1 DM without microalbuminuria and healthy people. The MDRD equation systematically underestimates glomerular filtration rate (GFR) when eGFR >60 ml/min/1.73m^2^ and overestimates GFR when eGFR <20 ml/min/1.73m^2^ and in patients with nephrotic syndrome [46], [47]. Besides, the MDRD equation was developed in whites and African Americans. Thus, it may not be accurate across racial groups because of differences among races in creatinine generation.

There were several limitations to our study. The number and interval of serum creatinine measurements varied in each patient. However, in order to decrease the chance of incomplete observation of change in renal function, we excluded patients with less than three eGFR measurements during the follow-up period and those patients with follow-up time less than 6 months. In addition, the majority of our patients were treated chronically with antihypertensive medications. For ethical reasons, we did not withdraw these medications. Hence, we could not exclude the influence of antihypertensive agents on our findings. Third, although P wave parameters can provide a simple and cheap method for detecting patients at risk of left atrial enlargement and left ventricular diastolic and systolic dysfunction, it cannot completely replace echocardiography. Finally, renal function impairment could yield a worse renal outcome and lead to biased results. However, to decrease this bias, we included the variable of baseline eGFR in the multivariate model.

In conclusion, our results demonstrate that PWdisperC >63.0 ms and PWdurMaxC >117.9 ms were associated with progression to renal end point. In addition, increased PWdisperC and PWdurMaxC were independently associated with progression to renal end point of ≥25% decline in eGFR. Screening patients by means of PWdisperC and PWdurMaxC on 12 lead ECG may help identify a high risk group of rapid renal function decline.
